# A web-based system to determine risk of investment in international rail construction projects

**DOI:** 10.1038/s41598-023-34358-7

**Published:** 2023-05-19

**Authors:** Ting Yuan

**Affiliations:** 1 School of Architecture and Civil Engineering, Xihua University, 9999 Hong Guang Avenue, Pidu District, Chengdu, 610039 Sichuan People’s Republic of China; 2 Research Center for Social Development and Social Risk Control, Sichuan Provincial Key Research Base of Philosophy and Social Sciences, No. 29, Wangjiang Road, Chengdu, 610064 Sichuan People’s Republic of China

**Keywords:** Civil engineering, Scientific data

## Abstract

Manual evaluation of investment risk make results and solutions are not timely. The objective of the study is to explore intelligent risk data collecting and risk early warning of international rail construction. First, this study has identified risk variables by content mining. Second, risk thresholds are calculated by the quantile method based on data from 2010 to A.D. 2019. Third, this study has developed risk early warning system by the gray system theory model, the matter-element extension method and the entropy weight method. Fourth, the risk early warning system is verified using Nigeria coastal railway project in Abuja. This study found that: (1) the framework of the developed risk warning system contains a software and hardware infrastructure layer, a data collection layer, an application support layer, and an application layer. (2) 37 investment risk variables are recognized; (3) 12 risk variables thresholds intervals are not equally divided between 0 and 1, the others are evenly distributed; (4) based on the application of Nigeria coastal railway project in Abuja, the system verification results are consistent with real situations, which is shown that risk early warning system is reasonable and feasible. These findings offer a good reference for intelligent risk management.

## Introduction

**Figure 1:**
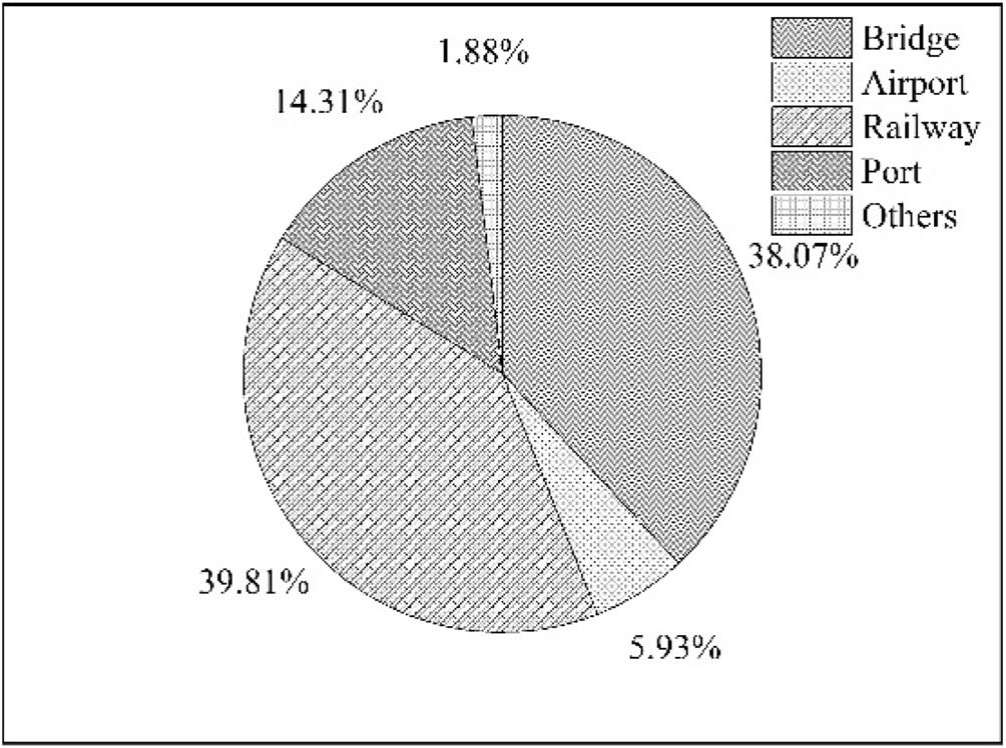
International construction projects invested by Chinese construction enterprises.

Chinese construction enterprises have given high priorities to rail constructions in the Belt and Road Initiatives countries^1^. According to the database of the Heritage Foundation, from 2005 to A.D. 2019, Chinese construction enterprises have participated in a total of 478 international transportation projects with $249.25 billion. These projects contained 239 bridges with $94.88 billion, 48 airport projects with $14.78 billion, 117 railway projects with $99.22 billion, 72 ports with $35.68 billion, and two others with $4.69 billion. It is obvious that Chinese construction enterprises have made enormous efforts and commitments to invest in rail constructions based on the Belt and Road Initiatives, which is shown in Fig. 1.

However, accidents occur frequently in international rail construction projects managed by Chinese construction enterprises, which have led to many economic losses because of their unpredictable investment environments, complex construction methods, and the divergent goals of local stakeholders^2^. For example, from 2005 to A.D. 2019, there were a total of 290 failed international investment cases by Chinese enterprises, which were distributed in 89 countries around the world. Among them, the failed railway projects involved $28,360 Millions. Therefore, the investment risk of international rail constructions needs to be explored so that reasonable measures can be taken. There is a general agreement that continuous monitoring of the investment risk are necessary during international rail construction.

It is difficult for Chinese construction enterprises to manually conduct risk evaluation and risk early warning based on a massive amount of data and multisource information. Such an undertaking requires rich professional knowledge and experience, is time consuming and has poor credibility in practice because of difficulties in handling large quantities of changing and multisource data^3^. Although previous studies have been conducted to manually evaluate investment risk in international construction projects^4,5^, in most cases, the results and solutions are limited to risk assessment management^6,7^. For example, Akunyumu^8^ has used questionnaire survey to recognized ten critical risk variables, which hold the ideal that unstable currency exchange rates, inflation, design changes, high-interest rate, budget overrun are important for managers. Face many risk information, some researchers regard that the framework of tacit knowledge externalization in international construction projects are key tools to manage risk^9^. However, risk knowledge of international construction projects are massive, if the manager want to get the information timely, this study thinks that automated risk early warnings by multisource information are necessary^10^. In addition, risk early warnings obtained by mathematical models and visual inspections of investment risks have become a necessary tool for risk analyses^11^.

Therefore, in order to develop a successfully automated risk early warning system, this study mainly focuses the following research questions: (1) how to design the risk early warning system? (2) in the risk early warning system, which risk variables can be used to conduct risk early warning? (3) how does the system integrate risk data to realize risk threshold analysis and risk early warning? (4) how is the effect of the risk early warning system designed in this study.

In the study, “Literature review” section reviews the relative literatures on investment risk of international construction projects. “Research methods of the risk early warning system in international rail construction project” section presents the methods used in investment risk early warning for international rail construction. “Results of the risk early warning system” section presents the results of system design and application of the system in Nigeria coastal railway project in Abuja. “Conclusion” section presents the conclusions. “Policy implications” section is Policy implications. “Limitation and future direction of research work” section concerns the limitation and future direction of research work.

## Literature review

### Investment risk assessment of international rail construction

Because investment accidents can cause significant economic losses in international rail construction, various state-of-the-art (SOTA) models published in recent 2 years have attempted to recognize and assess investment risk in the preconstruction phases of a project^12,13^. For example, some efforts to calculate investment risk and opportunity ratings have used the improved Multi-Attribute Decision Making Method (MADM)^14^. Other efforts have been made to develop financial risk assessments of risk events in international projects through stress testing. A structural equation model (SEM) was developed for incorporating political risk assessment into investment control^15^. Multivariate interrelationships among political risks were explored by factor analysis^16^. An analytic network process was used to develop a natural risk assessment of international airport projects for the location selection problem, which considered social, technical, economic, environmental and political criteria. The Monte Carlo simulation is a new way to assess the investment risk effects of international rail construction projects and is based on the probability distributions of the characteristics of data fluctuations in the past decade^1^. Although these SOTA models can evaluate the investment risk of international construction projects^17,18^, they can’t deal with multisource information immediately and they must process some of the data manually.

Meanwhile, most studies have paid attention to qualitative data analysis of investment risk during the construction phase. Previous research has used questionnaires and structural equation models to assess the investment risks of infrastructure projects in Central Asia in terms of social and political stability, institutions, and economic policies. Questionnaires and structural equation models are also common tools to assess the impact of the religious and cultural diversity risks of overseas projects in 33 countries on the performance of infrastructure projects from 1990 to A.D. 2014. The analytic hierarchy process is used to measure international project investment risks and medium and long-term investment opportunities from the dimensions of political environment, economic conditions, social environment, culture, religion, and climate. Some researchers hold the ideal that risks of the overseas water supply infrastructure based on questionnaires contain contract design, water prices, taxation, political stability, financing risks and water demand forecasts were key risks. A survey and the fuzzy comprehensive evaluation method is employed to fully consider the four dimensions of the risk values of land acquisition risk, commercial risk, operational risk and political risk of infrastructure projects to determine the overall risk of the project.

Although these risk assessment methods are available in international construction projects^19^, they have some shortcomings because they do not consider quantitative multisource information and large amounts of quantitative data^20,21^. These methods often focus on the investment risk state of a single component and qualitative analysis. As a result, the reliability and accuracy of investment risk early warnings cannot be ensured, and global automated investment risk assessment is impossible. Automatic data-driven approaches may be a useful tool for investment risk management of international construction projects.

### Data fusion in international construction projects

Data fusion is the process of using different sources to extract useful data and knowledge, improve reliability and reduce ambiguity^22,23^. The data fusion structure in risk warning systems can be divided into several phases: data collection, data analysis and decision making. Although each phase has its own target, function and limitations, the combination of different phases has been regarded as a useful way to analyze difficult and comprehensive problems^24,25^.

Previous studies indicate that data fusion in risk warning systems has become an area of intense interest that uses data visual methods to design risk assessment frameworks. In terms of visual risk, radial risk mapping has been used to graph international project risk during the bidding and proposal processes to select projects with the least risk^26^. Considering the weights and desired risk confidence level and tracking the changes in risk variation by experts’ reviews are regarded as reliable tools to monitor, track, and control the potential impacts of risk events^27^.

However, previous researches mainly pay attentions to visual risk assessment results, and overlook the reliability and accuracy of quantitative data source and data fusion steps. Risk warning systems can benefit from data fusion by improving and reducing ambiguity.

## Research methods of the risk early warning system in international rail construction project

**Figure 2:**
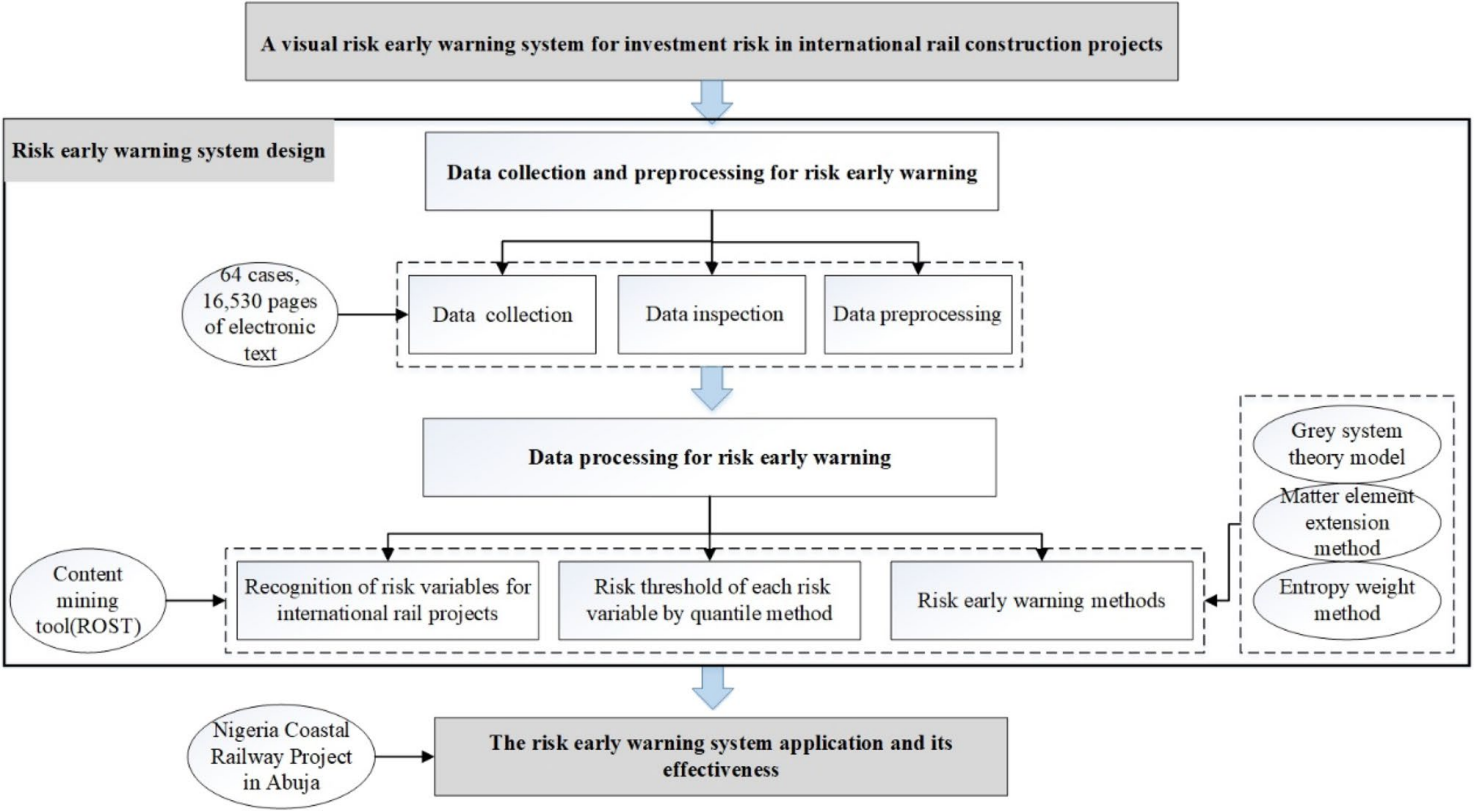
Research framework of the study.

In the risk early warning process, there are two distinct phases, risk early warning system design, the risk early warning system application and its effectiveness. Among them, risk early warning system design contains data collection, data processing. The detailed information has been represented in Fig. 2.

### Data collection and preprocessing for risk early warning

**Figure 3:**
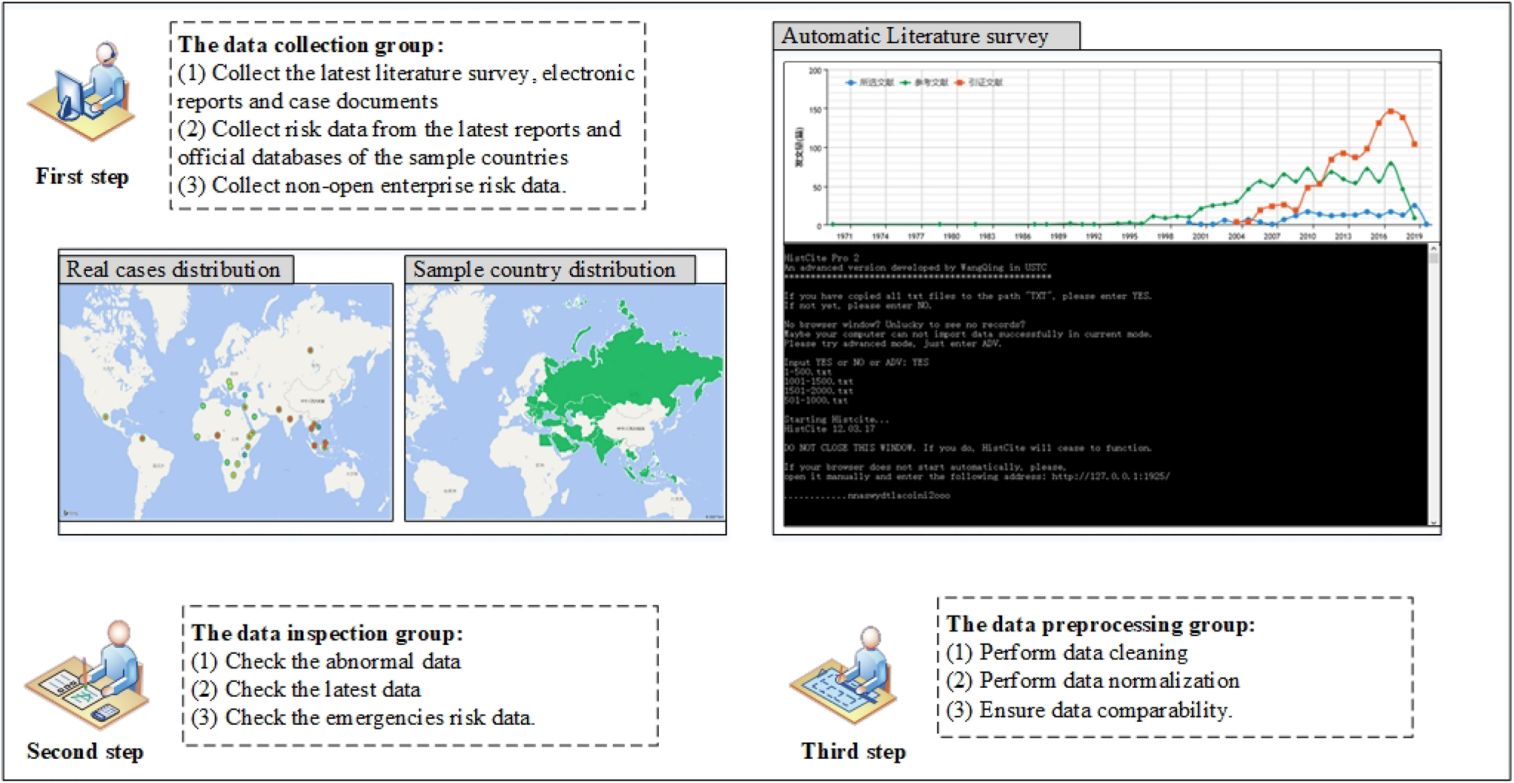
Information collection and preprocessing in the risk early warning.

Figure 3 represents information collection and preprocessing in the risk early warning system. It contains three steps, namely the data collection, the data inspection, the data preprocessing. The information of each part is detailed below.

The data collection group collects the context information from project audit reports, project cases, country (region) guides for foreign investment and cooperation, relevant literature into a project database in the form of an electronic text. The volume of the electronic text in the database is approximately 16,530 pages, and the number of real rail project cases is approximately 64, which are distributed in 38 countries including Malaysia, Thailand, Indonesia, Saudi Arabia, Philippines, Bangladesh, UAE, Cambodia, Sri Lanka, Kyrgyzstan, Kazakhstan, etc. All authorized users can not only search project details by keywords but can also scan relevant data. In addition, relevant countries’ economic and legal policies are also made available as electronic texts in the database.

There are the data inspection group and the data preprocessing group. On the one hand, the investment risk early warning system requires the data inspection group to check the abnormal data, the latest data and the emergency data. On the other hand, the literature data, report data and project case data need to first be interpreted and then normalized for further data fusion. The data preprocessing group needs to clean and normalize the risk early warning data to improve the comparability of the data. The detailed information has been represented in Fig. 3.

### Data processing for risk early warning

**Figure 4:**
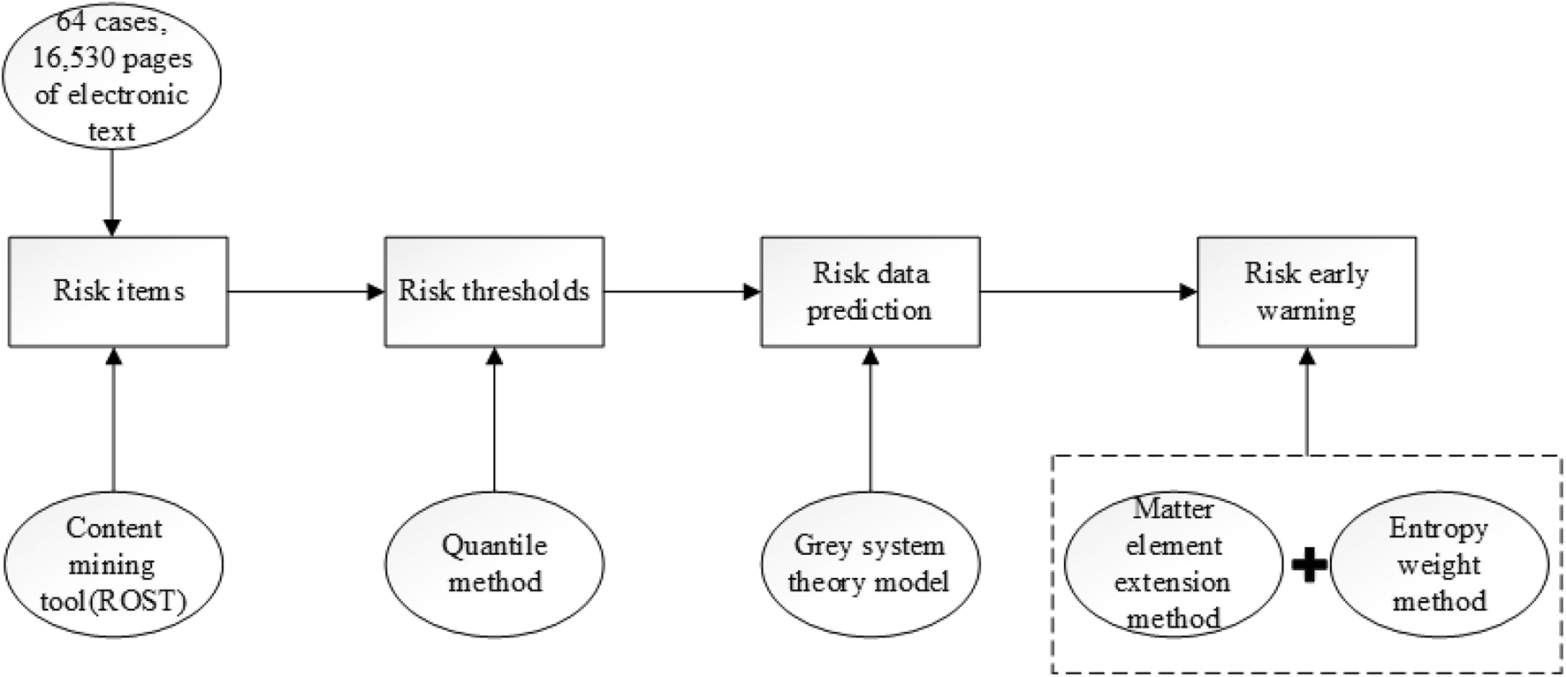
Data processing for risk early warning in the study.

There are several steps can be used to complete the data processing. (1) the system recognizes risk variables of international rail construction according to 64 cases, 16,530 pages of electronic text by content mining, which can be realize by the ROST CM6.0 software (ROST content-mining system version 6.0), which is a word-segmentation software used in content analysis. (2) the system conducts the risk early warning by the gray system theory model, the matter-element extension method and the entropy weight method. (3) the system gives the risk early warning levels of a specific investment risk, a specific risk category and an overall risk level. Figure 4 represents the data processing steps.

#### Recognition of risk variables for international rail projects

ROST content mining software can quantitatively organize, index, retrieve and utilize textual materials. Qualitative and quantitative analysis of risk variables based on ROST content mining software has been used in the study. This study has collected various electronic texts including literature, project audit reports, project cases, and country (region) guides for foreign investment and cooperation. The ROST content mining software has been used to extract the variables that appear most frequently in these electronic texts, which are regarded as risk variables. The advantage of the ROST content mining software is that it has the characteristics of dealing with massive information by intelligent and objective method. Moreover, the ROST content mining software can draw convincing universal conclusions from digital materials, and it can reduce the subjective influence of expert interviews and questionnaire surveys.

#### Exploring risk threshold of each risk variable

Different risk variables have different risk benchmarks in different countries^28^. In the study, the risk threshold is calculated by the quantile method and reflect the risk fluctuation features in different countries. The advantage of the method is that it can analyze a large amount of data simply and reasonably, reducing the vagueness and subjectivity of expert opinions^29^. The investment risk data came from many official reports around the world, and the data volume was large. Therefore, the quantile method can improve the efficiency of data processing. The detailed steps are presented as follows^30^:

This study has normalized each investment risk variable by the formula (1):

1 $$ Y_{ij} = \frac{{maxX_{ij} - X_{ij} }}{{maxX_{ij} - minX_{ij} }} $$

where ${X}_{ij}$ represents the initial risk data of the risk $i$ in the year $j$; $min{X}_{ij}$ represents the minimum data of risks; $max{X}_{ij}$ represents the maximum data of risks; and ${Y}_{ij}$ represents the normalized data of the risk.

Based on the normalized data of each investment risk, this study used the quantile method to divide each risk into 5 intervals, that is, $q=5$. The details have been represented in the formula (2):

2 $$ { }I_{ik} = Y_{ij} /q $$

where ${Y}_{ij}$ is sorted from small to large; ${I}_{ik}$ represents quantile data $k$ of the risk $i$.

#### Investment risk early warning methods of international rail construction projects

The gray system theory model, matter-element extension method and entropy weight method were used to conduct the risk early warning of investment risk. The following outlines the detailed steps.

First, the gray system theory model was implemented to predict the risk data of each risk variable in the near future. The advantage of the method is that it can analyze linear problems and solve nonlinear problems. The investment risks of international rail construction projects are complicated and contain linear and nonlinear problems. The basic principle of the gray system theory model is that it predicts risk data based on real data from the past, which can reduce subjectivity^31^. Meanwhile, compared with artificial neural networks, the data analysis results are more stable. The detailed steps of the gray system theory model are presented as follows formula (3), formula (4) and formula (5):

3 $$ \begin{aligned} X^{\left( 0 \right)} & = \left\{ {X^{\left( 0 \right)} \left( 1 \right),X^{\left( 0 \right)} \left( 2 \right), \ldots \ldots ,X^{\left( 0 \right)} \left( n \right)} \right\} \\ { }X^{\left( 1 \right)} & = \left\{ {X^{\left( 1 \right)} \left( 1 \right),X^{\left( 1 \right)} \left( 2 \right), \ldots \ldots ,X^{\left( 1 \right)} \left( n \right)} \right\} \\ & = \left\{ {X^{\left( 1 \right)} \left( 1 \right),X^{\left( 1 \right)} \left( 1 \right) + X^{\left( 0 \right)} \left( 2 \right), \ldots \ldots ,X^{\left( 1 \right)} \left( {n - 1} \right) + X^{\left( 0 \right)} \left( n \right)} \right\} \\ \end{aligned} $$

where ${X}^{\left(0\right)}\left(n\right)$ represents the initial risk data of risk X in year n.

$$ X^{\left( 0 \right)} \left( k \right) + aX^{\left( 1 \right)} \left( {\text{k}} \right) = {\text{b}},\;{\text{namely}},\;{ }\frac{dx}{{dt}} + ax = b $$

where $$\widehat{a}={(\mathrm{a},\mathrm{ b})}^{T}$$, $$\widehat{a}={({B}^{T}B)}^{-1}{B}^{T}Y$$,

4 $$ \begin{aligned} & {\text{B}} = \left[ {\begin{array}{*{20}c} { - \frac{1}{2}\left( {X^{\left( 1 \right)} \left( 1 \right) + X^{\left( 1 \right)} \left( 2 \right)} \right)} & 1 \\ { - \frac{1}{2}\left( {X^{\left( 1 \right)} \left( 2 \right) + X^{\left( 1 \right)} \left( 3 \right)} \right)} & 1 \\ { \ldots \ldots } & { \ldots \ldots } \\ { - \frac{1}{2}\left( {X^{\left( 1 \right)} \left( {n - 1} \right) + X^{\left( 1 \right)} \left( n \right)} \right)} & 1 \\ \end{array} } \right] \\ & Y_{m} = \left[ {\begin{array}{*{20}c} {X^{\left( 0 \right)} \left( 2 \right)} \\ {X^{\left( 0 \right)} \left( 3 \right)} \\ { \ldots \ldots } \\ {X^{\left( 0 \right)} \left( n \right)} \\ \end{array} } \right] \\ \end{aligned} $$

The predicted data are calculated using the following Eq. (5):

5 $$ \hat{X}^{\left( 1 \right)} \left( {k + 1} \right) = \left( {X^{\left( 0 \right)} \left( 1 \right) - \frac{b}{a}} \right)e^{ - k} + \frac{b}{a} $$

where k = 1, 2,…, n; − a is the development coefficient; and b is the gray effect. If − a < 0.35, then the prediction accuracy is better; if 0.35 ≤ − a < 0.5, then the prediction accuracy is qualified; if 0.5 ≤ − a < 0.65, then the prediction accuracy is average; and if − a ≥ 0.65, then the prediction accuracy does not meet the standard.

Second, the matter-element extension method was used to analyze the relationship between the risk threshold and the predicted risk data in the near future and to obtain the risk level of the risk variable. The advantage of the method is that it can take the features of the risk threshold into risk level prediction, which makes the prediction more accurate for countries around the world^32^. The detailed steps are presented as follows:

The evaluation matter matrix is calculated as formula (6):

6 $$ R = \left[ {\begin{array}{*{20}c} N & {\begin{array}{*{20}c} {\begin{array}{*{20}c} {r_{1} } & {x_{1} } \\ {r_{2} } & {x_{2} } \\ \end{array} } \\ {\begin{array}{*{20}c} \ldots & \ldots \\ {r_{m} } & {x_{m} } \\ \end{array} } \\ \end{array} } \\ \end{array} } \right] $$

where $\mathrm{R}$ represents the evaluation matter matrix; $\mathrm{N}$ represents matter; ${r}_{m}$ represents investment risks; and ${x}_{m}$ represents the predicted risk data.

Next, the classical matter-element matrix is calculated as formula (7):

7 $$ R_{p} = \left[ {\begin{array}{*{20}c} {N_{p} } & {\begin{array}{*{20}c} {\begin{array}{*{20}c} {r_{1} } & {x_{0j1} } \\ {r_{2} } & {x_{0j2} } \\ \end{array} } \\ {\begin{array}{*{20}c} \ldots & \ldots \\ {r_{m} } & {x_{0jm} } \\ \end{array} } \\ \end{array} } \\ \end{array} } \right] = \left[ {\begin{array}{*{20}c} {N_{0j} } & {\begin{array}{*{20}c} {\begin{array}{*{20}c} {r_{1} } & {\left[ {a_{0j1} ,b_{0j1} } \right]} \\ {r_{2} } & {\left[ {a_{0j2} ,{ }b_{0j2} } \right]} \\ \end{array} } \\ {\begin{array}{*{20}c} \ldots & \ldots \\ {r_{m} } & {\left[ {a_{0jm} ,{ }b_{0jm} } \right]} \\ \end{array} } \\ \end{array} } \\ \end{array} } \right] $$

where ${R}_{p}$ represents the matter-element matrix when the risk is at the $p$ risk level; ${N}_{0j}$ represents the risk factor at level j; and $\left[{\mathrm{a}}_{0jm},{\mathrm{b}}_{0jm}\right]$ represents the range of predicted risk data of risk m at level j.

For the risk level prediction, the following Eq. (8) are used:

8 $$ \begin{aligned} & k_{j} \left( {x_{m} } \right) = \frac{{p\left( {x_{m} ,x_{0jm} } \right)}}{{p\left( {x_{m} ,x_{pm} } \right) - p\left( {x_{m} ,x_{0jm} } \right)}} \\ & {\text{p}}\left( {{\text{x}}_{{\text{m}}} ,{\text{x}}_{{0{\text{jm}}}} } \right) = \left| {{\text{x}}_{{\text{m}}} - \frac{{{\text{a}}_{{0{\text{jm}}}} + {\text{b}}_{{0{\text{jm}}}} }}{2}} \right| - \frac{1}{2}\left( {{\text{b}}_{{0{\text{jm}}}} - {\text{a}}_{{0{\text{jm}}}} } \right) \\ & {\text{p}}\left( {{\text{x}}_{{\text{m}}} ,{\text{x}}_{{{\text{pm}}}} } \right) = \left| {{\text{x}}_{{\text{m}}} - \frac{{{\text{a}}_{{{\text{pm}}}} + {\text{b}}_{{{\text{pm}}}} }}{2}} \right| - \frac{1}{2}\left( {{\text{b}}_{{{\text{pm}}}} - {\text{a}}_{{{\text{pm}}}} } \right) \\ \end{aligned} $$

where j = 1, 2,…, s.

If ${k}_{j}\left({x}_{m}\right)>0$, then the predicted risk data meet the risk threshold of level j, and the larger the value, the closer it is to level j. If ${k}_{j}\left({x}_{m}\right)=0$, then the predicted risk data belong to the boundary point of level j. For ${k}_{j}\left({x}_{m}\right)<0$, the predicted risk data do not belong to level j.

For comprehensive risk assessment results, detailed formula (9) is presented below.

9 $$ K_{j} \left( R \right) = \mathop \sum \limits_{k = 1}^{m} w_{k} k_{j} \left( {x_{k} } \right) $$

Specifically, ${w}_{k}$ represents the importance of each investment risk. The entropy weight method was selected to calculate the importance of risks. The advantage of the method is that it can calculate the importance based on objective information, which can reduce human uncertainty^33^. The detailed steps are presented as follows^34^:

Entropy is calculated as follow formula (10):

10 $$ H_{j} = - \frac{{\left( {\mathop \sum \nolimits_{i = 1}^{m} f_{ij} lnf_{ij} } \right)}}{lnm} $$

where ${f}_{ij}=\frac{{b}_{ij}}{\sum_{i=1}^{m}{b}_{ij}}$, ${b}_{ij}$ represents the value of each risk variable.

Risk importance is calculated as follow formula (11):

11 $$ w_{j} = \frac{{\left( {1 - H_{j} } \right)}}{{\left( {n - \mathop \sum \nolimits_{j = 1}^{n} H_{j} } \right)}} $$

where ${w}_{j}$ represents the weight of the risk.

Finally, based on the principle of maximum relevance in extension theory, $\mathrm{k}\left(p\right)=max{K}_{j}(R)$, where $\mathrm{k}\left(p\right)$ is the result of the risk level.

The implementation of the comprehensive fusion model makes investment risk early warning possible in a simple way. The output of these models comprises five signals^35^, which are used in investment risk early warning decisions, and correspond to five investment risk early warning levels, which are represented by five different colors. Detailed information is presented in Table 1.

**Table 1:** Investment risk level and risk early warning signals.

Risk level	Signals	Descriptions
I	Green	The risk value is at the smallest level of the risk threshold, which represents the lowest level of risk
II	Orange	The risk is at a relatively small level of the risk threshold, which represents a relatively low level of risk
III	Yellow	The risk value is at a medium level of the risk threshold, which represents a medium level of risk
IV	Red	The risk value is at a relatively high level of the risk threshold, which represents a relatively high level of risk
V	Purple	The risk value is the highest level of the risk threshold, which represents the highest level of risk

### Methods of the risk early warning system application and its effectiveness

In order to verify the effectiveness of the risk early warning system, this study has selected the Nigeria coastal railway project in Abuja as the real case to conduct the application. The criteria for case selection are as follows. (1) Nigeria is the most populous country in Africa and is rich in oil and energy. Nigeria is also a country with more than 250 ethnic groups and 36 states, and is the leader of the economic community of China and African countries. It is a typical cooperation representative of construction projects between China and Africa. (2) Chinese construction enterprises and Nigerian railway construction have many opportunities for cooperation. With the rapid economic development in Nigeria, there are more than 3800 km of existing railways, 3505 km of which are narrow-gauge monorail lines. It is obvious that Nigeria has a large demand for railway construction. (3) In recently, the China-Africa Construction Corporation and the Federal Ministry of Transport of Nigeria have signed a framework contract for the Nigeria coastal railway project in Abuja for $11.97 billion, which is the representative project for China and Africa.

This study would use the risk early warning system to collect data and conduct risk early warning of Nigeria coastal railway project in Abuja. Then, this study has collected the qualitative risk information of Nigeria construction market from Economic and Commercial Office of the Embassy of the People’s Republic of China in the Federal Republic of Nigeria. Finally, the system results would be compared with results of qualitative risk information in the above official website to verify the effectiveness of the risk early warning system in the study.

### Ethics approval

This article does not contain any studies with human participants performed by the author.

## Results of the risk early warning system

### Results of risk early warning system design

**Figure 5:**
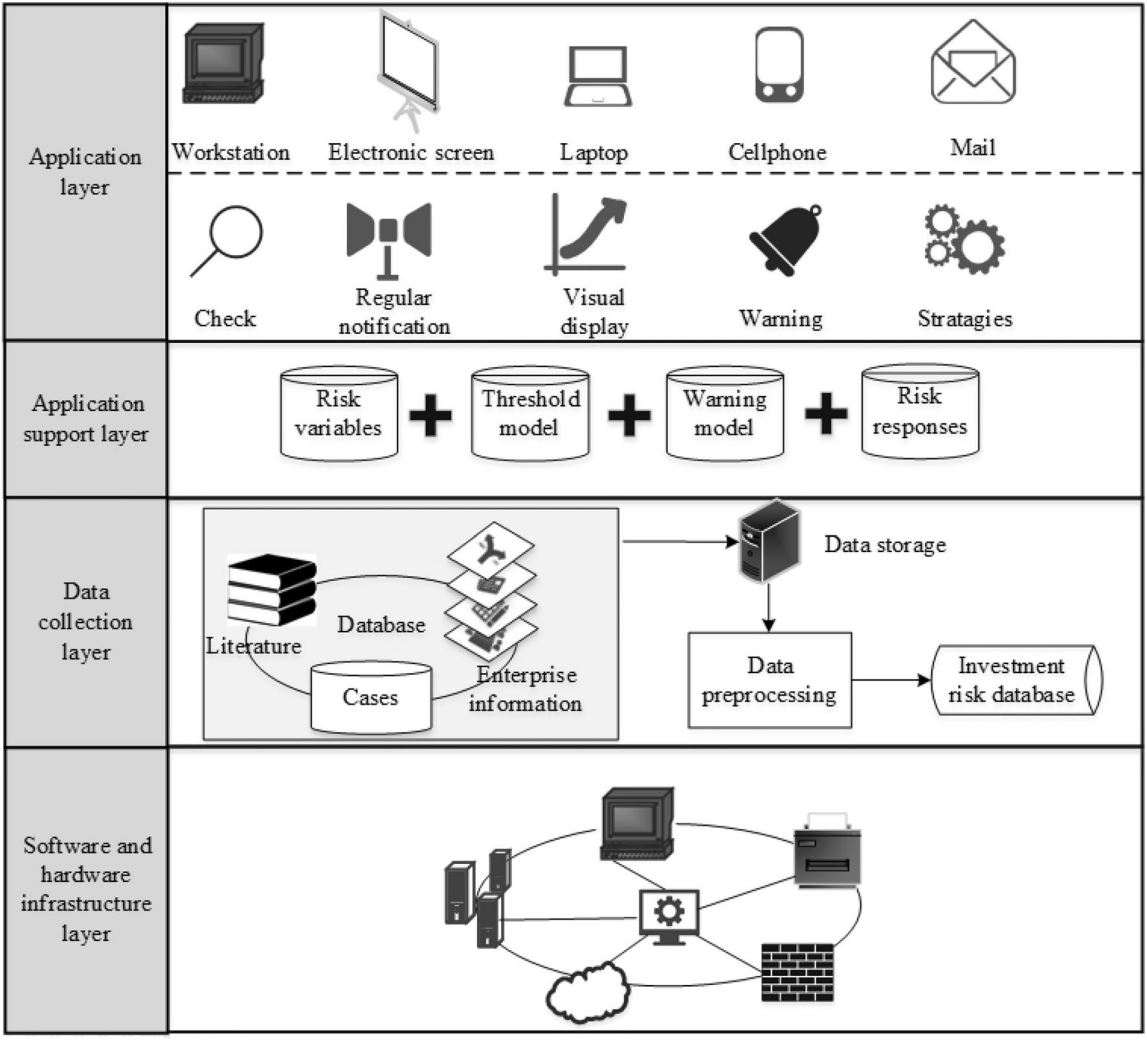
Framework of the investment risk early warning system.

The risk early warning system for investment risk in international rail construction projects is a decision support system that contains the responsibility and workflow of all operators, experts, and decision makers, and it can control all risk information and databases. To receive risk early warning information from everywhere, it is necessary to develop an automatic network system. The framework of the developed system is composed of four typical levels, namely, a software and hardware infrastructure layer, a data collection layer, an application support layer, and an application layer, which are shown in Fig. 5.

In this system, the software and hardware are responsible for building the network. The data collection layer is responsible for collecting and preprocessing data from the multisource information uploaded in real time. The application support layer is the core component containing the risk variables, the threshold model, the risk early warning model containing the GM model, the matter-element extension method and the entropy weight method to automatically conduct the investment risk early warning. The application layer allows users to receive warning signals and exchange information.

Considering the convenience and characteristics of the system structure defined above, a software-based system was built in this research, which users can obtain access by their own computers. The major development tools of the system include Microsoft Visual C++ 2015 and LabWindows_CVI 2017. The system was designed by a Windows 10 SDK 1803 server, which is a high-performance server, and the speed of the processors is 2.80 GHz. The system offers high calculation ability to store, retrieve and analyze data.

### Results of risk early warning system application in Nigeria coastal railway project in Abuja

#### Results of investment risk variables in the risk early warning system

The investment risk early warning system has recognized 37 risk variables. These risk variables can be divided into 6 categories, namely, political risk, economic risk, legal and policy risk, public safety risk, sociocultural risk, engineering market risk. This study has selected words with a frequency of more than 50% in the electronic texts as risk variables for overseas railway projects. According to the results, employee’s work attitude is the most frequent, namely, 90.21%. The detailed information has been represented in the Table 2.

**Table 2:** Risk variables and their frequency in electronic texts.

Code	Risk variables	Frequency (%)
Political risk	The relationship between the investing country and the country where the project is located (R1)	51.23
	Political stability (R2)	56.87
	Corruption control degree (R3)	77.21
	Government credit level (R4)	66.35
	Administrative efficiency (R5)	58.21
Economic risk	Difficulty of financing (R6)	59.33
	Tax ratio (R7)	61.21
	Inflation (R8)	66.54
	Exchange rate fluctuations (R9)	71.01
	Debt ratio (R10)	65.89
Legal and policy risk	Policy change (R11)	66.03
	Construction permit processing time (R12)	58.42
	Strict work permit system (R13)	60.99
	Material clearance time (R14)	57.39
	Difficulty of land acquisition (R15)	60.02
	Human resources employment restrictions (R16)	82.31
	Exit cost of investing enterprise (R17)	55.21
	Strict technical standards for railway projects (R18)	68.43
	Strict HSSE standards (R19)	79.23
	Contract dispute handling efficiency (R20)	63.22
Public safety risk	Social Security (R21)	78.21
	Terrorism (R22)	89.21
	Epidemic (R23)	78.36
	Natural disaster (R24)	65.23
Sociocultural risk	Cultural distance (R25)	88.42
	Employee’s work attitude (R26)	90.21
	Staff skill training level (R27)	74.14
	Public attitude (R28)	79.36
	Educational level of residents (R29)	63.05
Engineering market risk	Transport infrastructure demand (R30)	70.89
	Capacity of existing railway projects (R31)	65.31
	Urban population growth rate (R32)	73.24
	Availability of electricity (R33)	58.42
	Credit of local suppliers (R34)	66.98
	Number of local suppliers (R35)	84.32
	Number of competitive companies (R36)	67.05
	Contract execution efficiency (R37)	77.34

#### Results of investment risk threshold of each risk variable in the risk early warning system

To analyze investment risk threshold of each risk variable, the investment risk data of Nigeria coastal railway project in Abuja needs to be preprocessed before risk warning is launched. The normalized data has been represented in the Table 3.

**Table 3:** The normalized data of Nigeria coastal railway project in Abuja.

Variable	2010	2011	2012	2013	2014	2015	2016	2017	2018	2019
R1	0.357	0.274	0.230	0.307	0.400	0.377	0.440	0.305	0.258	0.355
R2	0.773	0.880	0.813	0.980	0.976	0.988	0.972	0.825	0.825	0.825
R3	0.921	0.956	0.955	0.970	0.985	0.930	0.914	0.929	0.920	0.920
R4	0.326	0.395	0.333	0.372	0.411	0.434	0.349	0.372	0.372	0.372
R5	0.006	0.026	0.055	0.057	0.000	0.066	0.029	0.051	0.048	0.048
R6	0.150	0.156	0.184	0.095	0.075	0.088	0.129	0.122	0.109	0.143
R7	0.662	0.800	0.855	0.759	0.862	0.917	0.821	0.710	0.710	0.710
R8	0.667	0.722	0.696	0.767	0.775	0.757	0.630	0.614	0.698	0.711
R9	0.996	0.996	0.996	0.996	0.996	0.995	0.994	0.993	0.993	0.993
R10	0.978	0.977	0.980	0.979	0.977	0.968	0.956	0.936	0.927	0.927
R11	0.575	0.640	0.526	0.605	0.675	0.623	0.522	0.601	0.601	0.601
R12	0.507	0.507	0.880	0.880	0.837	0.851	0.851	0.851	0.845	0.845
R13	0.250	0.250	0.250	0.250	0.250	0.250	0.250	0.250	0.250	0.250
R14	0.101	0.223	0.331	0.079	0.058	0.036	0.058	0.086	0.065	0.058
R15	0.491	0.476	0.531	0.599	0.613	0.790	0.790	0.794	0.809	0.817
R16	0.958	0.965	0.895	0.937	0.965	0.951	0.902	0.930	0.902	0.888
R17	0.476	0.476	0.476	0.476	0.476	0.476	0.476	0.476	0.476	0.476
R18	0.326	0.261	0.217	0.138	0.101	0.152	0.203	0.094	0.080	0.058
R19	0.667	0.667	0.667	0.667	0.667	0.667	0.667	0.667	0.667	0.667
R20	0.531	0.622	0.671	0.469	0.322	0.427	0.406	0.315	0.231	0.287
R21	0.162	0.141	0.106	0.049	0.092	0.127	0.155	0.134	0.134	0.134
R22	0.184	0.136	0.061	0.041	0.075	0.088	0.109	0.102	0.102	0.102
R23	0.000	0.010	0.019	0.030	0.040	0.051	0.062	0.062	0.062	0.074
R24	0.949	0.999	0.447	0.989	1.000	0.991	0.993	0.989	0.945	0.980
R25	1.000	1.000	1.000	1.000	1.000	1.000	1.000	1.000	1.000	1.000
R26	0.901	0.960	0.937	0.857	0.874	0.897	0.879	0.933	0.933	0.933
R27	0.489	0.604	0.612	0.698	0.676	0.576	0.532	0.482	0.367	0.288
R28	0.158	0.281	0.171	0.041	0.144	0.103	0.137	0.151	0.151	0.151
R29	0.188	0.188	0.188	0.188	0.188	0.188	0.188	0.188	0.188	0.188
R30	0.057	0.121	0.177	0.092	0.064	0.064	0.071	0.071	0.128	0.085
R31	0.217	0.217	0.287	0.240	0.248	0.225	0.225	0.271	0.000	0.016
R32	0.504	0.501	0.497	0.493	0.488	0.483	0.478	0.473	0.468	0.468
R33	0.245	0.360	0.323	0.356	0.353	0.311	0.409	0.338	(0.451)	0.338
R34	0.719	0.617	0.555	0.641	0.703	0.727	0.734	0.789	0.789	0.789
R35	0.206	0.382	0.603	0.522	0.324	0.309	0.309	0.456	0.456	0.456
R36	0.579	0.500	0.364	0.664	0.593	0.486	0.521	0.521	0.521	0.521
R37	0.830	0.830	0.830	0.830	0.366	0.631	0.631	0.631	0.750	0.777

**Figure 6:**
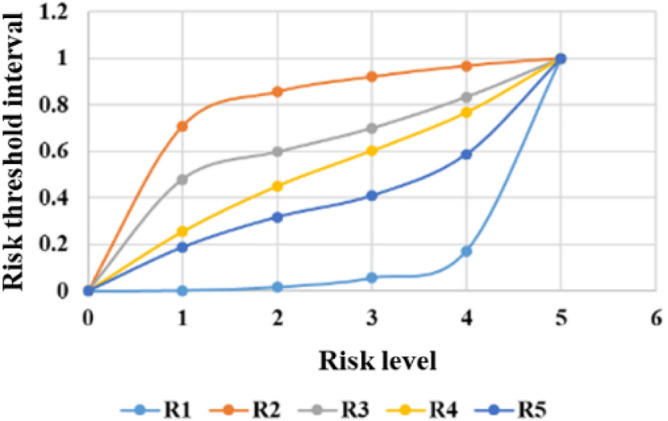
Political risk threshold range.

**Figure 7:**
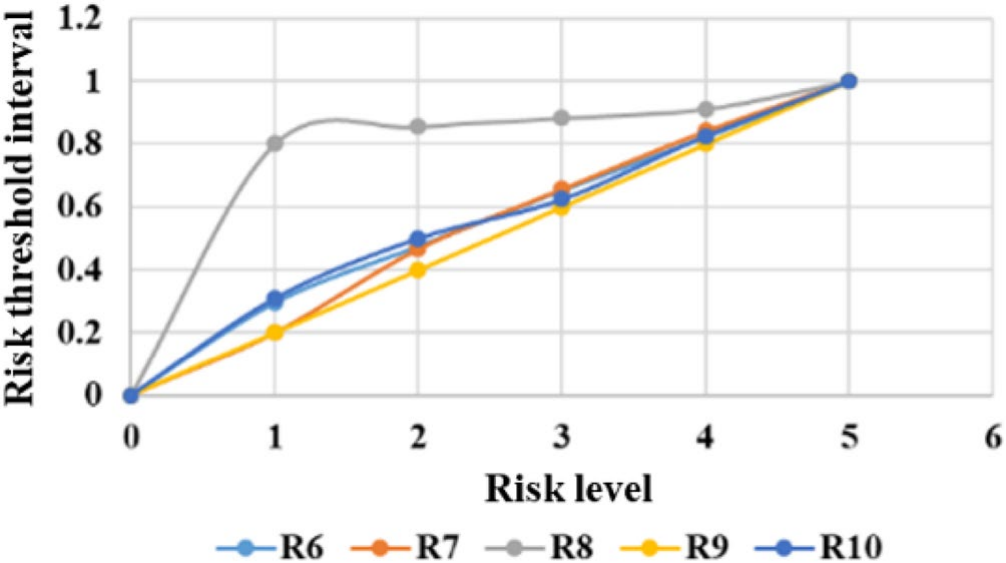
Economic risk threshold range.

**Figure 8:**
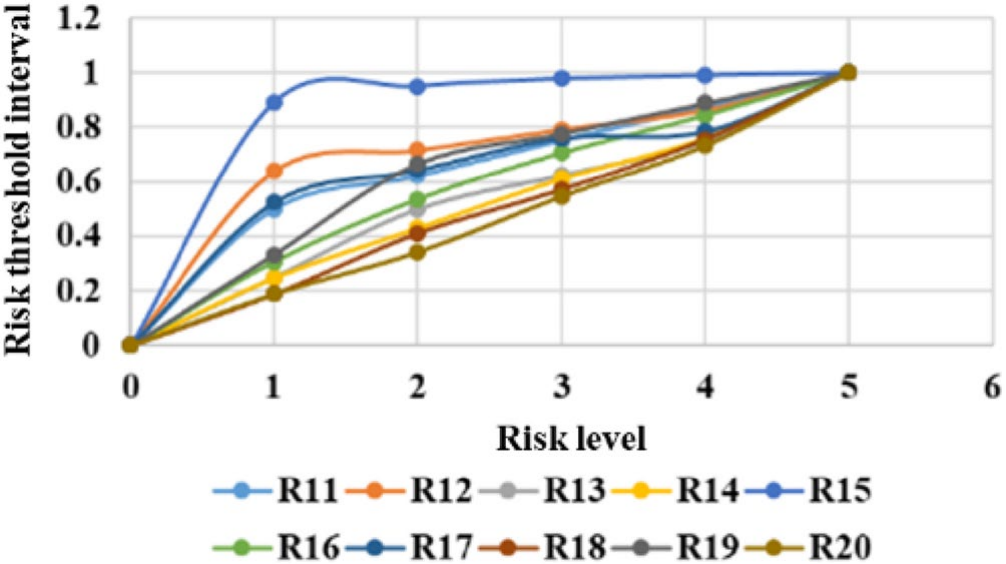
Legal and policy risk threshold range.

**Figure 9:**
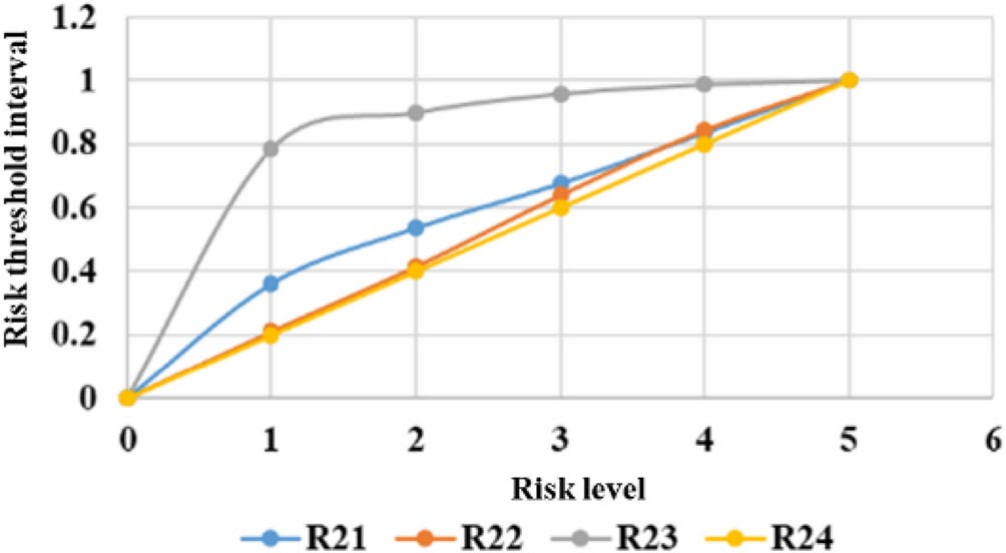
Public safety risk threshold range.

**Figure 10:**
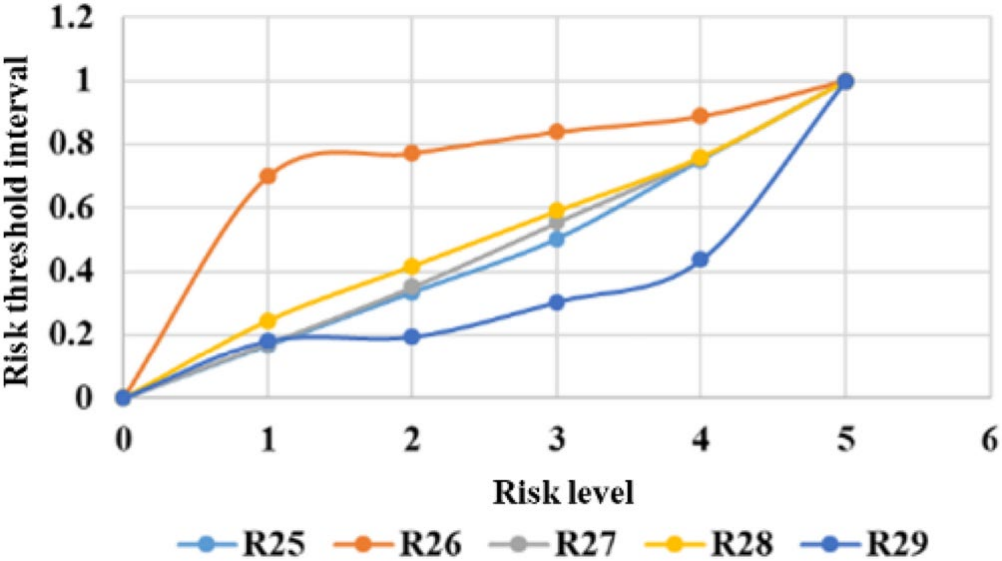
Sociocultural risk threshold range.

**Figure 11:**
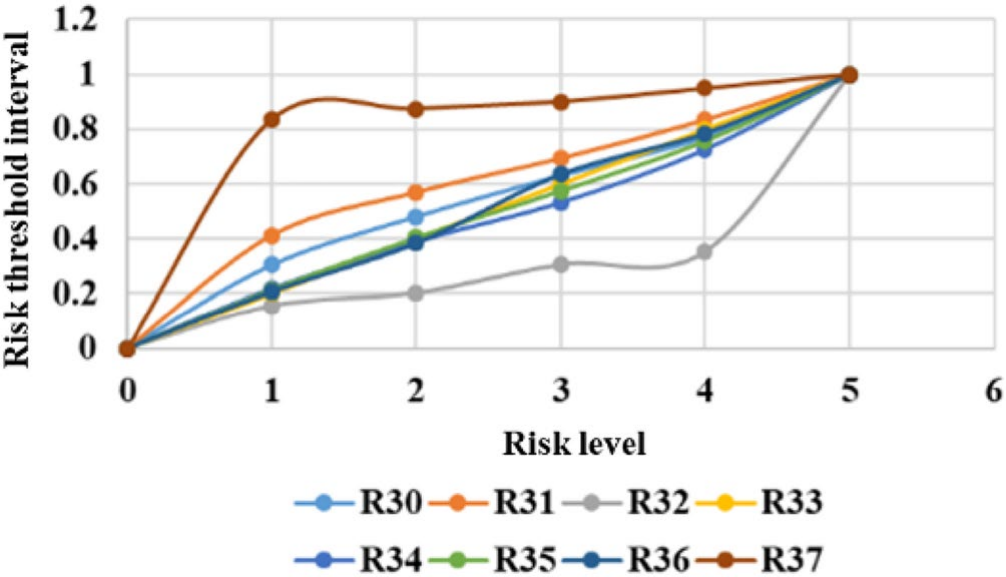
Engineering market risk threshold range.

The threshold limits for classifying different risk levels are calculated by the quantile method. The system can produce comprehensive statistical graphs to visualize the time series of the measurement data, rate of change, and threshold limits, as shown in Figs. 6, 7, 8, 9, 10, and 11. The detailed information of the results about threshold limit has been represented in Table 4. Furthermore, if the threshold limit is exceeded, then the levels of investment risk and the risk maps are automatically presented on the system screen. To ensure accuracy, when the threshold limit is exceeded, the risk early warning is repeated to confirm the results depending on the situation.

**Table 4:** The detailed information of the results about threshold limit.

Variables	Min	1	2	3	4	Max
R1	0.000	0.003	0.018	0.057	0.174	1.000
R2	0.000	0.709	0.857	0.921	0.968	1.000
R3	0.000	0.480	0.598	0.700	0.834	1.000
R4	0.000	0.255	0.450	0.603	0.768	1.000
R5	0.000	0.189	0.318	0.410	0.588	1.000
R6	0.000	0.296	0.476	0.653	0.823	1.000
R7	0.000	0.200	0.468	0.657	0.841	1.000
R8	0.000	0.801	0.855	0.882	0.912	1.000
R9	0.000	0.200	0.400	0.600	0.800	1.000
R10	0.000	0.310	0.501	0.625	0.826	1.000
R11	0.000	0.500	0.623	0.754	0.877	1.000
R12	0.000	0.639	0.717	0.793	0.864	1.000
R13	0.000	0.250	0.500	0.625	0.750	1.000
R14	0.000	0.247	0.430	0.612	0.763	1.000
R15	0.000	0.891	0.951	0.978	0.990	1.000
R16	0.000	0.308	0.537	0.708	0.846	1.000
R17	0.000	0.524	0.643	0.762	0.786	1.000
R18	0.000	0.188	0.412	0.572	0.758	1.000
R19	0.000	0.333	0.667	0.778	0.889	1.000
R20	0.000	0.189	0.343	0.547	0.734	1.000
R21	0.000	0.362	0.537	0.676	0.835	1.000
R22	0.000	0.211	0.415	0.639	0.844	1.000
R23	0.000	0.785	0.899	0.958	0.987	1.000
R24	0.000	0.200	0.400	0.600	0.800	1.000
R25	0.000	0.168	0.336	0.504	0.752	1.000
R26	0.000	0.700	0.771	0.839	0.888	1.000
R27	0.000	0.173	0.351	0.554	0.755	1.000
R28	0.000	0.247	0.418	0.590	0.760	1.000
R29	0.000	0.182	0.195	0.305	0.437	1.000
R30	0.000	0.305	0.481	0.631	0.773	1.000
R31	0.000	0.413	0.570	0.694	0.835	1.000
R32	0.000	0.156	0.204	0.307	0.355	1.000
R33	0.000	0.200	0.400	0.600	0.800	1.000
R34	0.000	0.219	0.391	0.533	0.727	1.000
R35	0.000	0.214	0.404	0.575	0.757	1.000
R36	0.000	0.207	0.384	0.636	0.786	1.000
R37	0.000	0.836	0.875	0.901	0.949	1.000

#### Results of risk early warning in Nigeria coastal railway project in Abuja by the system

**Figure 12:**
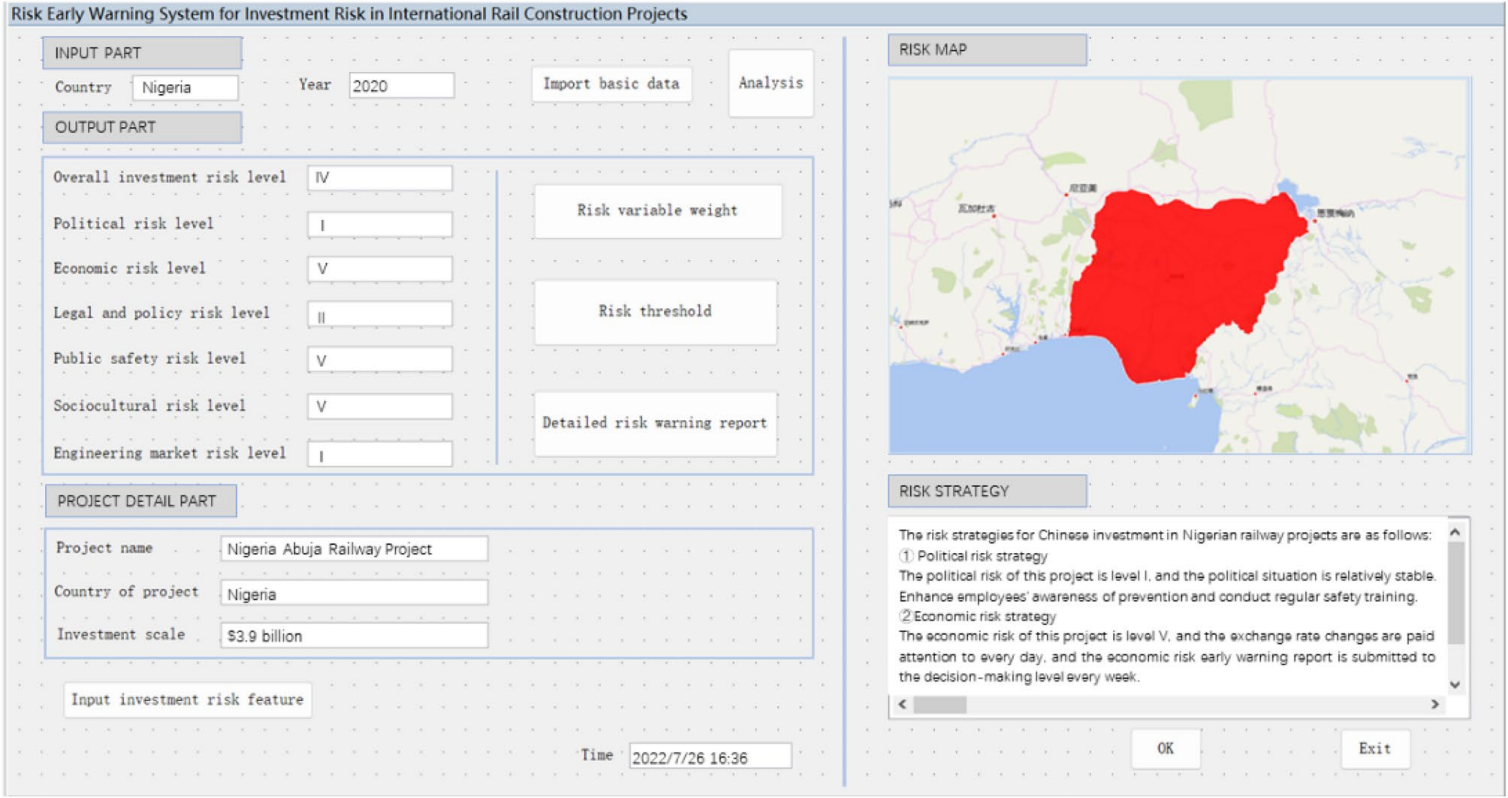
Risk early warning in Nigeria coastal railway project in Abuja by the system.

The application of the risk early warning system to Nigeria coastal railway project in Abuja, unlike previous studies, which have only paid attention to risk assessment, indicates that the system makes it possible to analyze a massive amount of data quickly and systematically. In the risk early warning system, risk variables with different risk levels are automatically presented in the regular warning reports, as shown in Fig. 12. In the Nigerian case, 37 early warning variables divided into 6 categories and different risk level descriptions were made for the visual investment risk early warning system. The results of the risk early warning system indicate that the comprehensive investment risk level is IV, the political risk level is I, the economic risk level is V, the legal and policy risk level is II, the public safety risk level is V, the sociocultural risk level is V, and the rail engineering market risk level is I. When the risk map of Nigeria is not green, all the possible investment risk countermeasures checked by the expert group and the decision makers are automatically represented in the system.

#### Results of risk early warning system effectiveness

To explore the effects of the risk early warning system, this study compares qualitative risk information of Nigeria construction market from Economic and Commercial Office of the Embassy of the People’s Republic of China in the Federal Republic of Nigeria with the risk levels of the risk early warning system. Detailed information of the comparative analysis for supporting the effectiveness of the proposed risk early warning system are presented in Table 5. These results can prove the reasonability of the system.

**Table 5:** Comparative results of qualitative risk information of Nigeria construction market with risk levels of the risk early warning system.

Number	Qualitative risk information of Nigeria construction market	System application result	Application effect
1	The political situation stabilized, and President Buhari was re-elected	Political risk level is I	Corresponding
2	Abuja rail project faces tight operating capital issues	Engineering market risk level is I	
3	It is difficult for Chinese construction enterprises to remit funds freely	Economic risk level is V	Corresponding
4	Abuja-Kaduna rail passenger service suspended due to measures to prevent COVID-19	Public safety risk level is V	Corresponding
5	The official exchange rate and the parallel market exchange rate have a difference of 50%	Economic risk level is V	Corresponding
6	The tracks between Rijana and Dutse stations of the Abuja-Kaduna Railway were blown up by explosives planted by unknown persons	Public safety risk level is V	Corresponding
7	Nigeria’s debt levels have grown year-on-year, and the federal government needs more loans to fund critical infrastructure such as roads, railways and electricity	Economic risk level is V	Corresponding
8	In terms of land acquisition procedures, market liquidity, transparency, and certainty are poor	Legal and policy risk level is II	Corresponding
9	Nigeria has not yet established an intellectual property law, and relevant regulations are included in the commercial law	Legal and policy risk level is II	Corresponding
10	Due to ethnic and religious conflicts, Nigeria’s public security is a significant threat. At the end of July 2020 A.D., a total of 808 people were kidnapped, and there is a tendency for this practice to spread	Public safety risk level is V	Corresponding
11	There were seven large-scale strikes and several nationwide strikes from 2013 to A.D. 2019, which seriously affected the international investment efficiency of enterprises	Sociocultural risk level is V	Corresponding
12	The education level of residents is relatively low, the illiteracy rate is close to 46%, and the skilled labor force is insufficient	Sociocultural risk level is V	Corresponding

## Conclusion

The investment risks in international rail construction cannot be managed by single source information, models and techniques^36^. This kind of complicated project contains multisource and systematic information, which needs to be considered before making decisions in relation to investment risk early warning management. Traditional manual and single management models published in recent 2 years such as AHP, stress testing, SEM focus on subjective risk data and expert experience, which ignore the importance of multiple objective data sets and intelligent tools.

Compared with these SOTA models, the risk early warning system containing comprehensive models in the study has considered the development of intelligent technologies. The main contributions of the study are as follows: (1) in the risk early warning system, risk variables have been recognized by massive quantitative data; (2) different risk threshold for each index has been calculated according to its numerical fluctuation characteristics over the last decade; (3) the system can realize risk early warning level of each risk variable and recognize critical risk variables automatically.

The significances of the study are as follows: (1) it is possible for the risk early warning system in international rail construction to collect risk data, risk early warning and visualize warning results using the internet. (2) the system allows enterprises to receive investment risk early warnings automatically based on the comprehensive fusion model without a massive amount of subjective knowledge. (3) the risk early warning system can improve efficiency and accuracy, and provide intelligent investment risk management. (4) with the development of international project scales, enterprises can use it to grasp investment risk information accurately and in advance to make reasonable decisions. The proposed intelligent and collaborative risk early warning system is an attractive tool for international enterprises in investment risk management for international rail construction.

## Policy implications

This study has offered many policy implications. First, this study has used the risk early warning system to recognize risk variables. It is believed that international project managers in Nigeria should pay more attentions to the important risk variables, such as public safety risk and social-cultural risk. The second policy implication concerns the necessary of further intelligent research in risk management. Given the massive risk data of international construction projects, this study believes that data collection, data process and results’ visual representation must consider intelligent tools to improve the efficiency of the decision-making.

## Limitation and future direction of research work

There are also several limitations to this study and future directions for the risk early warning system of international rail construction projects. For research limitations, the amount of multisource data in the system is limited, and improved knowledge and additional cases can be obtained through data mining technologies. For future direction of the study, it is necessary to improve the function of the risk early warning system of international rail construction project by dynamic user requirements.

## Data Availability

All data, models, or code that support the findings of this study are available from the corresponding author upon reasonable request. (List item: Investment risk data of Nigerian railway projects invested by Chinese enterprises).
